# Agricultural land management and rural financial development: coupling and coordinated relationship and temporal-spatial disparities in China

**DOI:** 10.1038/s41598-024-57091-1

**Published:** 2024-03-19

**Authors:** Maogang Gong, Ruichao Xi, Yuxi Qi, Xizhe Wang, Pengsheng Sun, Lingling Che

**Affiliations:** 1 College of Economics, Shandong University of Technology, Zibo, 255000 Shandong People’s Republic of China; 2 Marxism Sinicization Research Center, Shandong University of Technology, Zibo, 255000 Shandong People’s Republic of China; 3 Business School, Shandong University of Technology, Zibo, 255000 Shandong People’s Republic of China

**Keywords:** Agricultural land management, Rural finance, Coupling coordination, Time–space difference, Socioeconomic scenarios, Sustainability

## Abstract

The integrated development of agricultural land and finance not only promotes rural financial innovation and breaks the bottleneck of agricultural financing but also facilitates agricultural land transfer and scaled operations. This leads to the advancement of the effective growth of contemporary agriculture. The reform of the 'separation of three rights' in agricultural land promotes land circulation, which, in turn, offers an institutional guarantee for the tandem development of rural finance and agricultural land management. This paper measures the comprehensive development index of agricultural land management and rural finance in 30 provinces of China from 2005 to 2020. In light of this, it calculates the degree of coupling and coordination between China's agricultural land management and rural financial development. The Dagum Gini coefficient, kernel density, and the Moran index were used to analyze regional differences and patterns of agglomeration. The study found that the degree of coupling coordination between China's agricultural land management and rural finance is increasing annually. However, there remains a significant gap in achieving high-quality coupling. Notably, the growth rate of rural financial development exceeds that of agricultural land management, and hypervariable density is a major source of regional variation. There is polarization in the coupled development of farmland management and rural finance. Provinces in the eastern and central regions tend to be located in the high–high agglomeration (H–H) in terms of the level of development of agricultural land and financial integration, while the western region tends to fall in low–low aggregation (L–L).

## Introduction

The inherent weaknesses of agriculture, characterized by lengthy investment cycles, high business risks, low and uncertain returns, and limited finances creating a persistent bottleneck in China's agricultural growth. The delineation and protection of the "three rights of ownership, contracting, and management" in agricultural property have been instrumental in addressing the financial constraints impeding modern agricultural development and laying a robust foundation for farmland financing. The "separation of rights" not only clarifies and fortifies the property rights over agricultural land, prompting land transfer and large-scale operations, but also enhances investment efficiency and returns, thereby stimulating substantial capital demand. Concurrently, the "land management rights" serve as collateral security, mitigating information asymmetry, enhancing the creditworthiness of agricultural operators, and boosting the willingness of financial institutions to fund agricultural endeavors.

The process of coordinated development of farmland management and rural finance is to break through the bottleneck of land fragmentation management through land circulation and realize large-scale management of rural land based on the rural land property right system and the mortgage loan of farmland management right as an important carrier, thus providing conditions for the mortgage loan of management right^1,2^. At the same time, various mortgage loans of management rights are used to finance the circulation of rural land, improve the efficiency of factor allocation, and promote the efficient development of modern agriculture^3^. The organic integration of rural land and rural finance is an important way to enhance the endogenous power and ability of rural development and promote rural revitalization. This study defines the coordinated development of farmland management and rural finance as: from the perspective of system, the two as a whole realize the transition from dissonance to coordination; From the perspective of individuals, agriculture land management and rural finance promote each other, thus achieving a relative balance.

Based on the current problems of capital shortage and low factor allocation efficiency in the process of farmland management, this study has three main purposes, namely (1) to identify the relationship between agriculture land management and rural finance; (2) to determine feasible methods to achieve the coordinated development of agriculture land management and rural finance and provide policy recommendations; (3) to judge the regional differences in the coordinated development of agriculture land management and rural finance, and analyze the reasons for the differences. Therefore, this paper uses the entropy method to measure the agriculture land management index and rural finance development index respectively. The coupling coordination model is used to measure the absolute change of their coordinated development, and the coupling coordination type is divided into the advantage type of agriculture management (JY) and the advantage type of rural finance (JR), and the relative change of the two subsystems is explored. Then the provinces of China are divided into four regions, and the differences between regions and the sources of the differences are analyzed using the dagum Gini coefficient and the kernel density method. Finally, Moran's index is used to analyze the degree of spatial aggregation of coordinated development.

Against the background of the "separation of rights", this study combines agriculture land management with capital financing, which not only helps to solve the dilemma of effective inputs of agricultural production factors, but also is of great significance in promoting the in-depth integration of agricultural land management and rural finance, and provides the necessary data support for planning work. It proposes a development model that meets national conditions and local realities at the level of China as a whole, regions and provinces, so that the integration of agricultural land and financial integration can develop in a high-quality and coordinated manner, thus accelerating the development process of rural revitalisation.

In the rest of the paper, we provide a literature review in “Literature review”, in “Data and methods” we present the research methodology, data and sources, in “Results” we measure the degree of coupled coordination between agriculture management and rural finance and analyse the relative advantage, in addition we present the regional differences, spatial–temporal evolution and spatial aggregation of the coupled coordination, and in “Conclusions” we summarise the conclusions and make recommendations.

## Literature review

### Study on the development model for the integration of agricultural land management and rural finance

In China, the prevailing approaches to integrating farmland and finance encompass a variety of models, including farmland management right mortgages, farmland credit cooperatives, farmland trusts, farmland financial leasing, farmland securities, and land banks^4,5^. Notably, the farmland management right mortgage was initially designed to enhance loan accessibility for farmers by assigning collateral value to farmland, to enable farmers to obtain more credit funds^6,7^. This model has fostered a stable supply–demand relationship within the rural financial industry, thereby rendering the integration of farmland management and rural finance development a practical proposition^8,9^. Farmland credit cooperatives represent a hybrid organization that consolidates farmers' idle land into large-scale agricultural parcels, which are then transferred to new agricultural management entities^10^. Within the framework of agricultural land and finance integration, these cooperatives play a pivotal role in bolstering the agricultural sector^11^. They also deliver crucial financial services in support of supply-side structural reform^12^. Beyond these traditional models, innovative approaches have also emerged, including land title mortgages, agricultural value chain services, and mortgage securitization^9,13,14^^.^ These effective mechanisms further diversify the ways in which agricultural land and finance can be effectively integrated, thereby contributing to the evolution and sophistication of this critical sector.

### Study on factors affecting the integration and development of agricultural land management and rural finance

The validation of farmland rights is pivotal for the integration of agricultural land and financial development. Some scholars put forward emphasize that inadequacies in affirming and certifying agricultural land rights can severely limit farmers' access to loans, detrimentally affecting their immediate interests^15^. A well-crafted policy for confirming farmland rights can not only vitalize agricultural resources but also expand the scope of farmland transfer^16,17^. Further, a robust land transfer system is critical for the integrated development of farmland and finance^18^. Liang et al.^19^ pointed out that a sound farmland transfer mechanism can optimize the allocation of resources like land, loans, and labor, thereby facilitating the unified development of farmland and finance. In addition, increasing the construction of clean energy in rural areas and combining green finance with agriculture and rural areas can help promote the upgrading of rural industries^20–22^. The role of rural financial services is also crucial in this integration. Xia^23^ highlighted that a varied rural financial system can effectively distribute resources to the rural economy and society, which can address the diverse financial needs of farmers. Some scholars proposed that establishing a refined financial service mechanism for farmland could shift the focus of rural financial development policy towards financial support, thus fostering a cohesive development between farmland and finance^24,25^. This comprehensive approach underscores the necessity of well-defined farmland rights and efficient financial mechanisms for the sustainable integration of agricultural land and finance.

### Measurement study on the development of agricultural land and financial integration

In examining the integration of farmland and financial development, various approaches have been adopted to measure this relationship. Sarma^26^ introduced the Financial Inclusion Index (IFI), employing dimensions such as permeability, service accessibility, and usage utility. Building on this, Gupte et al.^27^ enhanced the IFI by incorporating factors like convenience and transaction costs, thereby broadening the scope of financial inclusion assessment for farmland. Wang et al.^28^ segmented the rural financial inclusion index into three subsystems: permeability, utility of use, and affordability. They explored the relationship of this index with income disparity and regional differences. Peng et al.^29^ focused on examining the inclusive financial development in developing the degree of cooperation between agricultural industrialization growth levels and financial accessibility, Further deepening the research, Zhang et al.^30^ evaluated the coordination and coupling level between integrated rural industrial development and inclusive finance. Their findings indicated significant regional disparities and a generally low coupling level between these systems. Fibæk et al.^31^ used the geospatial decision system to measure the spatial service scope of rural finance, Zhou et al.^32^ employed spatial panel modeling to probe the spatial correlation in rural financial inclusion.

Most of the existing research is on the model, influencing factors and measurement of the coordinated development of agriculture land management and rural finance. Few scholars have analyzed the regional differences, spatial evolution, and aggregation characteristics of coupling relationships. Therefore, it is necessary to conduct more in-depth research on the coupling relationship between agriculture land management and rural finance. The research results have important practical significance for further optimizing the allocation of rural production factors and realizing agricultural modernization.

## Data and methods

### Construction of the variable system

#### Agricultural land management index

A total of seven variables were selected to construct the evaluation index system for measuring the farmland management index. These include the arable land area and effective irrigated area, which are fundamental to farmland management^33^. The land transfer area indicates the extent of farmland management at an appropriate scale^34^. The crop planting area reflects the utilization of farmland^35^, while crop output per unit of land represents the efficiency of the output from the farmland^36^. Additionally, the number of large and medium-sized tractors for agricultural use, along with the number of farm implements accompanying these tractors, measure the elements of material equipment, representing the scale of land management^37^. All these variables positively contribute to the assessment.

#### Rural finance index

To construct the assessment index system for the rural financial index, a total of seven variables were selected. Firstly, the number of business locations and personnel of rural financial institutions illustrates the range and extent of services they offer^38^. Secondly, the volume of loans and agricultural loans issued by these institutions indicates the level of capital availability in rural areas. Lastly, the amount of deposits at rural financial institutions, savings accumulated by farmers, and income generated from agricultural insurance premiums reflect the degree of utilization of rural finance^39^.

In this paper, seven positive variables were selected from each of the two systems of agricultural land management and rural finance, totaling 14 variables. Table 1 displays the specific variables and units.

**Table 1:** Variables for evaluating the agricultural land management index and the rural finance index.

Setup	Variables	Unit (of measure)	Variables positive or negative
Agricultural land management index	Cropland area	Thousand hectares	+
	Land transfer area	Thousand hectares	+
	The area under crop cultivation	Thousand hectares	+
	Crop output per unit of land	Tonnes/thousand hectares	+
	Effective irrigated area	Thousand hectares	+
	Number of agricultural large and medium-sized tractors	Piece	+
	Number of implements for large and medium-sized tractors	Piece	+
Rural finance index	Number of business outlets of rural financial institutions	Piece	+
	Number of employees of rural financial institutions	Person	+
	Deposits in rural financial institutions	Billions yuan	+
	Loans from rural financial institutions	Billions yuan	+
	Agricultural loans from rural financial institutions	Billions yuan	+
	Farmers' savings	Billions yuan	+
	Agricultural insurance premium income	Million yuan	+

#### Variable data sources and descriptive statistics

**Figure 1:**
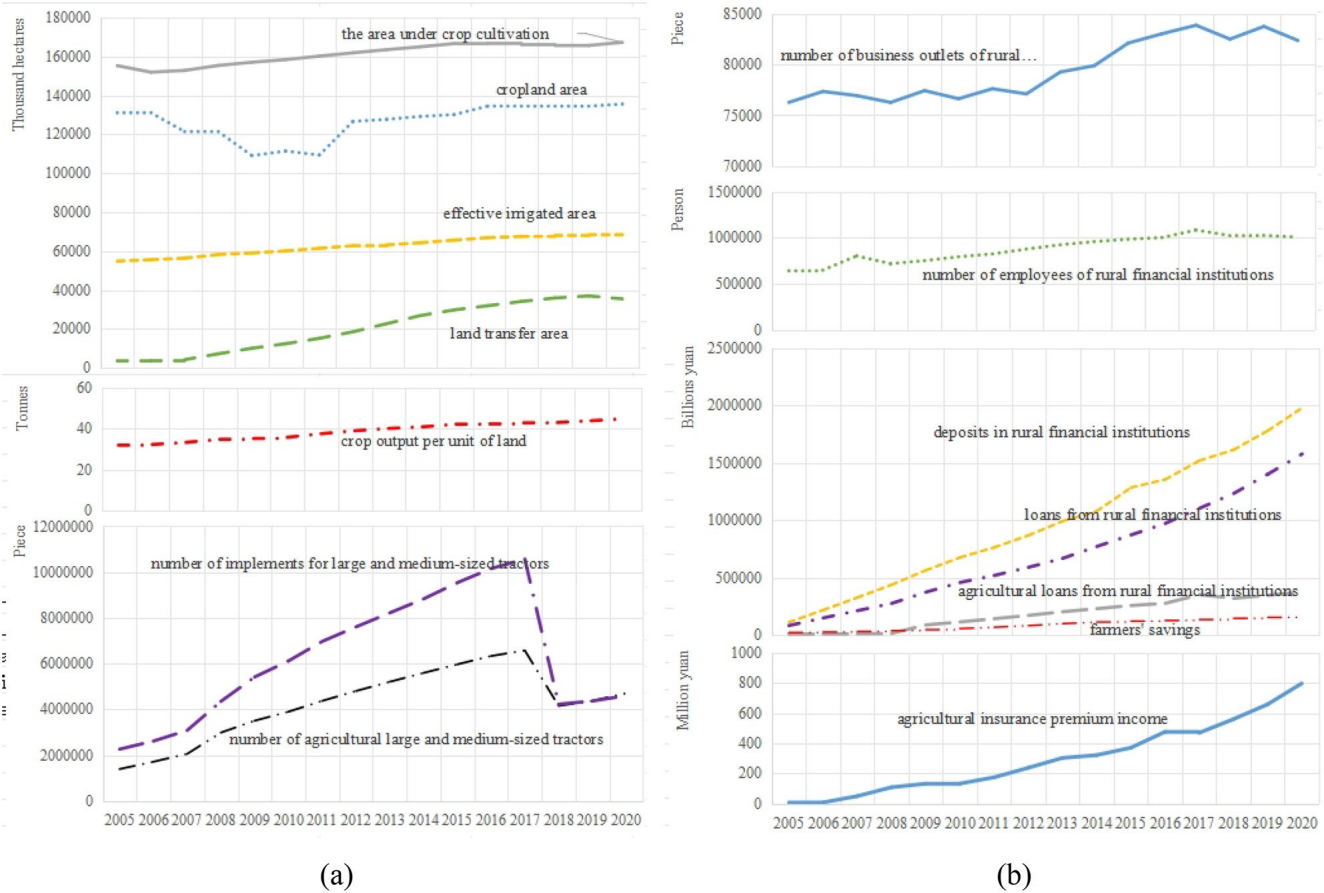
The trend of the variables. (**a**)Agriculture land management variables. (**b**)Rural finance variables.

The sample size of this paper is 30 provinces, including municipalities and autonomous regions across China, with the exception of Tibet, Hong Kong, Macao, and Taiwan, covering the period from 2005 to 2020. It comprises a total of 480 observations across 14 selected variables. The relevant data were sourced from various authoritative databases and publications. Among them, cropland area, the area under crop cultivation, crop output, effective irrigated area, number of agricultural large and medium-sized, number of implements for large and medium-sized, data from *China Statistical Yearbook* and statistical yearbooks of provinces and cities (2006–2021). land transfer area data from *China Rural Business Management Statistical Yearbook* (2006–2021); number of business outlets of rural financial, agricultural loans from rural financial data from *China Rural Financial Services Report* for the biennial years of 2008–2020 (published in every biennial year). number of employees of rural financial, deposits in rural financial institutions, loans from rural financial institutions, farmers' savings data from *China Financial Yearbook* (2006–2021), agricultural insurance premium income data from *China Insurance Yearbook* (2006–2021). Missing data are filled in using linear interpolation, Descriptive statistics of relevant variables are detailed in Table 2. The trend of the variables is shown in Fig. 1.

**Table 2:** Descriptive statistics of variables.

Variables	Mean value	Standard error	Minimum	Maximum	Median
Cropland area	4211.820	2969.098	184.190	16,184.300	4162.210
Land transfer area	685.557	817.370	6.013	4598.205	388.974
The area under crop cultivation	5372.895	3772.638	88.550	14,910.130	4925.525
Crop output per unit of land	38.797	30.825	1.293	138.080	33.988
Effective irrigated area	2086.072	1573.208	109.240	6177.590	1612.145
Number of agricultural large and medium-sized tractors	140,819.957	184,164.309	104.000	1,060,600.000	67,528.500
Number of implements for large and medium-sized tractors	205,985.622	297,049.281	156.000	1,449,600.000	56,679.000
Number of business outlets of rural financial institutions	2650.640	1678.622	312.000	6263.000	2404.000
Number of employees of rural financial institutions	29,240.163	20,400.063	58.000	144,251.000	26,190.000
Deposits in rural financial institutions	32,507.718	37,076.237	121.468	256,174.507	20,244.565
Loans from rural financial institutions	23,536.031	26,339.612	49.844	188,473.331	15,719.203
Agricultural loans from rural financial institutions	6171.022	7332.183	9.980	40,007.150	3404.830
Farmers' savings	3066.459	3028.170	24.530	15,914.320	2154.315
Agricultural insurance premium income	1003.447	1243.260	0.080	9873.110	578.100

### Research methods

#### Entropy method

The entropy method is an objective assignment method, which further determines the contribution of each indicator by the value of the information entropy of the indicator. Where i denotes the system, *j* denotes the first indicator, ${x}_{ij}$ denotes the original value, ${y}_{ij}$ denotes the standardised value, and ${w}_{ij}$ denotes the indicator weights which are determined using the entropy value method. The data is standardised and positive indicators with the following equation.

1 $${{\text{y}}}_{{\text{ij}}}=\frac{{{\text{x}}}_{{\text{ij}}}-{\text{min}}\left({{\text{x}}}_{{\text{ij}}}\right)}{{\text{max}}\left({{\text{x}}}_{{\text{ij}}}\right)-{\text{min}}\left({{\text{x}}}_{{\text{ij}}}\right)}$$

Calculate comprehensive evaluation indicators

2 $${{\text{U}}}_{{\text{i}}}=\sum_{{\text{j}}=1}^{{\text{n}}}{{\text{w}}}_{{\text{j}}}*{{\text{y}}}_{{\text{ij}}}$$

#### Coupled coordination degree model

This study measures the coupled coordination relationship between farmland management and rural finance. The coupled coordination degree model is a crucial tool for analyzing the relationships between systems.

3 $${\text{C}}=2{\left[{\left({{\text{U}}}_{1}*{{\text{U}}}_{2}\right)/\left({{\text{U}}}_{1}+{{\text{U}}}_{2}\right)}^{2}\right]}^{1/2}$$

4 $${\text{T}}=\mathrm{\alpha }{{\text{U}}}_{1}+\upbeta {{\text{U}}}_{2}$$

5 $${\text{D}}=\sqrt{{\text{C}}*{\text{T}}}$$

where ${{\text{U}}}_{1}$ is the value of the composite variable of farmland management, and ${{\text{U}}}_{1}$ is the comprehensive index value of rural finance; *C* is the coordination degree of agricultural land management system and rural financial system, *C* ∈ [0, 1], when *C* tends to 1, the two systems reach a "benign resonance", and when *C* tends to 0, the two systems reach a "disorder" between them.; *T* is the comprehensive evaluation index of farmland management system and rural financial system; $\mathrm{\alpha }$ and $\upbeta$ are coefficients to be determined, $\mathrm{\alpha }$ + $\upbeta$ = 1, considering that the two systems are equally important in this paper, so $\mathrm{\alpha }$=$\upbeta$=0.5. "D is the coupling degree of farmland management system and rural financial system.

Three categories exist for the degree of coupling coordination. stronger inter-system coupling coordination is indicated by a number that is closer to 1, and worse inter-system coupling coordination is indicated by a value that is closer to 0. Ten categories can be used to categorize the degree of coupling coordination, as Table 3 illustrates.

**Table 3:** Criteria for judging the degree of coordination of the coupling of agriculture land management and rural finance.

Degree of coupling coordination D	Scope D	Type of coupled coordination
Imbalance	[0, 0.1)	Extreme disorder
	[0.1, 0.2)	Severe disorder
	[0.2–0.3)	Moderate disorder
	[0.3, 0.4)	Mild disorder
Antagonism	[0.4–0.5)	On the verge of becoming dysfunctional
	[0.5, 0.6)	Sue for coordination
Coordination	[0.6–0.7)	Primary coordination
	[0.7, 0.8)	Intermediate level coordination
	[0.8–0.9)	Good coordination
	[0.9, 1.0)	Quality coordination

#### Dagum's Gini coefficient and its decomposition

To analyze the overall differences and the underlying reasons for differences in the coupled development of the two systems, this article employs the Dagum Gini coefficient to measure the regional differences in the coupled development of farmland management and rural finance. The Dagum Gini coefficient decomposes the total differences into three components: intra-regional differences, inter-regional differences, and hypervariable density. The contribution of hypervariable density is specifically attributed to the existence of interregional cross-over terms.

where *j* and *h* denote two regions.${n}_{j}, {n}_{h}$ denote the number of provinces within the corresponding regions, respectively ${o}_{ji}, {o}_{hr}$ denote the coupling coordination degree of the mth province within region j and the rth province within region h, respectively.$\overline{{{\text{o}} }_{{\text{i}}}}, \overline{{{\text{o}} }_{{\text{h}}}}$ are the means, respectively, and k is the number of regions is 4. If the two regions calculated are the same region, then *j* = *h*. *G* is the overall Dagum Gini coefficient, and ${{\text{G}}}_{{\text{w}}}$ is the intra-region variation contribution, and ${{\text{G}}}_{{\text{nb}}}$ is the inter-district variation contribution, and ${{\text{G}}}_{{\text{t}}}$ is the hypervariance density.

Calculation of the inter-regional Gini coefficient:

6 $${{\text{G}}}_{{\text{jh}}}=\frac{{\sum }_{{\text{m}}=1}^{{{\text{n}}}_{{\text{j}}}}\sum_{{\text{r}}=1}^{{{\text{n}}}_{{\text{h}}}}\left|{{\text{o}}}_{{\text{jm}}}-{{\text{o}}}_{{\text{hr}}}\right|}{{{\text{n}}}_{{\text{j}}}{{\text{n}}}_{{\text{h}}}\left(\overline{{{\text{o}} }_{{\text{j}}}}+\overline{{{\text{o}} }_{{\text{h}}}}\right)}$$

The Dagum Gini coefficient is further decomposed into three components:

7 $${\text{G}}={{\text{G}}}_{{\text{w}}}+{{\text{G}}}_{{\text{nb}}}+{{\text{G}}}_{{\text{t}}}$$

8 $${\text{G}}={\sum }_{{\text{j}}=1}^{{\text{k}}}{{\text{G}}}_{{\text{jj}}}{{\text{p}}}_{{\text{j}}}{{\text{s}}}_{{\text{j}}}+\sum_{{\text{j}}=1}^{{\text{k}}}\sum_{{\text{h}}\ne {\text{j}}}{{\text{G}}}_{{\text{jh}}}{{\text{p}}}_{{\text{j}}}{{\text{s}}}_{{\text{h}}}{{\text{Z}}}_{{\text{jh}}}+\sum_{{\text{j}}=1}^{{\text{k}}}{\sum }_{{\text{h}}\ne {\text{j}}}{{\text{G}}}_{{\text{jh}}}{{\text{p}}}_{{\text{j}}}{{\text{s}}}_{{\text{h}}}(1-{{\text{Z}}}_{{\text{jh}}})$$

where ${{\text{p}}}_{{\text{j}}}$ represents the ratio of the number of provinces within region *j* to the total number of provinces in the region, and ${{\text{S}}}_{{\text{h}}}, {{\text{S}}}_{{\text{j}}}$ represents the proportion of the coupling coordination between regions *h* and *j* to the coupling coordination of all provinces in the sample. ${{\text{Z}}}_{{\text{jh}}}$ represents the relative influence of the coupling coordination between region *j* and region *h*.

#### Kernel density estimation

Kernel density estimation is the use of a smooth kernel function to describe the dynamic trend of the spatiotemporal distribution of variables, this paper apply kernel density estimation to analyse the dynamic evolution of the coupling degree between farmland management and rural financial development in China, the expression of which is shown in Eq. (10). where ${{\text{n}}}_{{\text{j}}}$ is the number of sample observations in region j, K is the kernel density function, and H is the bandwidth.

9 $${{\text{f}}}_{{\text{j}}}({\text{o}})=\frac{1}{{{\text{n}}}_{{\text{j}}}{\text{h}}}\sum_{{\text{i}}=1}^{{{\text{n}}}_{{\text{j}}}}{\text{K}}(\frac{{{\text{c}}}_{{\text{ji}}}-{\text{c}}}{{\text{H}}})$$

#### Moran index

1. Global Moran's index
  The global Moran's index is used to determine whether the samples as a whole are spatially correlated, and the global Moran's index equation is as follows:
  10 $${\text{I}}=\frac{{\text{n}}}{{{\text{S}}}_{0}}\frac{\sum_{{\text{i}}=1}^{{\text{n}}}\sum_{{\text{j}}=1}^{{\text{n}}}{{\text{W}}}_{{\text{ij}}}({{\text{o}}}_{{\text{i}}}-\overline{{\text{o}} })({{\text{o}}}_{{\text{j}}}-\overline{{\text{o}} })}{\sum_{{\text{i}}=1}^{{\text{n}}}{({{\text{o}}}_{{\text{i}}}-{{\text{o}}}_{{\text{j}}})}^{2}}$$
  where I denotes the global Moran index, the ${{\text{o}}}_{{\text{i}}}$ and ${{\text{o}}}_{{\text{j}}}$ are the coupling coordination degrees of provinces i and j, respectively. ${{\text{W}}}_{{\text{ij}}}$ is the spatial weight matrix between provinces i and j. When two provinces and cities are not neighbouring, the ${{\text{W}}}_{{\text{ij}}}=0$ ; when the two provinces and cities are neighbouring, the ${{\text{W}}}_{{\text{ij}}}=1$ that ${{\text{S}}}_{0}$ is the aggregation of all spatial weights, and n is the number of provinces and cities under study.
  The global Moran index ranges between -1 and 1. A positive index (I > 0) suggests a positive spatial correlation, indicating that provinces and cities with similar levels of agricultural land and financial development tend to cluster together; the higher the value, the stronger this positive correlation. Conversely, a negative index (I < 0) points to a negative spatial correlation, meaning provinces and cities with differing development levels are grouped together, with stronger negative correlation indicated by smaller values. An index of zero (I = 0) signifies no spatial correlation, suggesting a random spatial distribution in the integration of agricultural land and financial development.
2. Local Moran's index
  The method is used to analyse the degree of spatial correlation between each region and its neighbouring regions, which can reflect spatial heterogeneity, explore whether the geospatial units belong to high-value agglomeration or low-value agglomeration, and visually represent them by drawing the Moran scatterplot. The Moran scatter plot consists of four quadrants, namely first quadrant (high–high agglomeration), second quadrant (low–high agglomeration), third quadrant (low–low agglomeration) and fourth quadrant (high–low aggregation). High–high aggregation represents that the region has a high level of development of agricultural land and financial integration and is surrounded by areas with a high level of development. High–low aggregation means that the region has a high level of development of agricultural land and financial integration and is surrounded by neighbouring regions with a low level of development of agricultural land and financial integration.The calculation formula is:
  11 $${{\text{I}}}_{{\text{i}}}={{\text{B}}}_{{\text{i}}}\sum_{{\text{j}}=1,\mathrm{ j}\ne 1}^{{\text{n}}}{{\text{W}}}_{{\text{ij}}}{{\text{B}}}_{{\text{j}}}$$
  ${{\text{B}}}_{{\text{i}}}$ and ${{\text{B}}}_{{\text{j}}}$ denote the normalised values of observations on spatial cells i and j respectively, and ${{\text{W}}}_{{\text{ij}}}$ is the spatial weight.

## Results

### Measuring the coupled and coordinated relationship

#### Measurement of the agricultural land management index and the rural financial development index

**Figure 2:**
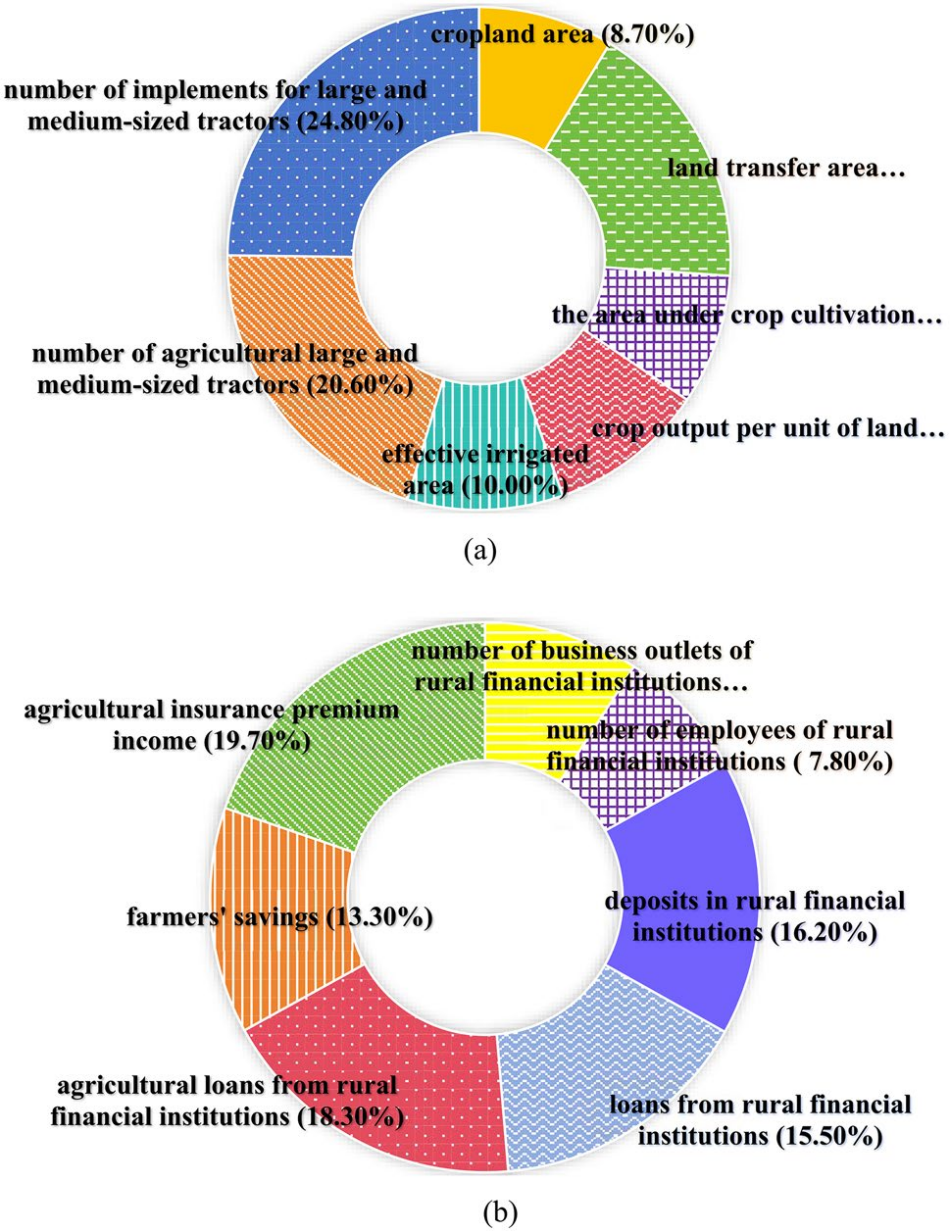
Variable weights. (**a**) Agricultural land management variable weights. (**b**) Rural finance variable weights.

Figure 2 shows the weights of the variables calculated using the entropy method. It shows that the weights of the area of arable land, the area under crop cultivation, the number of business outlets of rural financial institutions, and the number of employees at these institutions are 0.087, 0.084, 0.091, and 0.078, respectively. Each of these weights is below 0.1, indicating that the impact of these variables on their respective systems is relatively small. Conversely, the weights of the number of large- and medium-sized tractors for agricultural use, the number of farm implements compatible with these tractors, agricultural loans from rural financial institutions, and agricultural insurance premium income are significantly higher, being 0.206, 0.248, 0.183, and 0.197, respectively. As all these values exceed 0.18, these indicate a greater impact on their corresponding systems.

**Figure 3:**
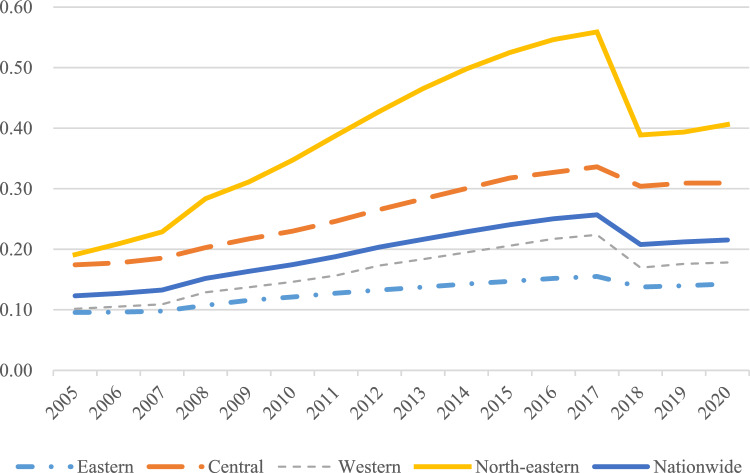
National and regional agricultural land management index.

1. Agricultural land management index
  The results of measuring the agricultural land management index across various regions of the country are shown in Fig. 3, while Fig. 4 shows the mean value of this index in 30 provinces.

**Figure 4:**
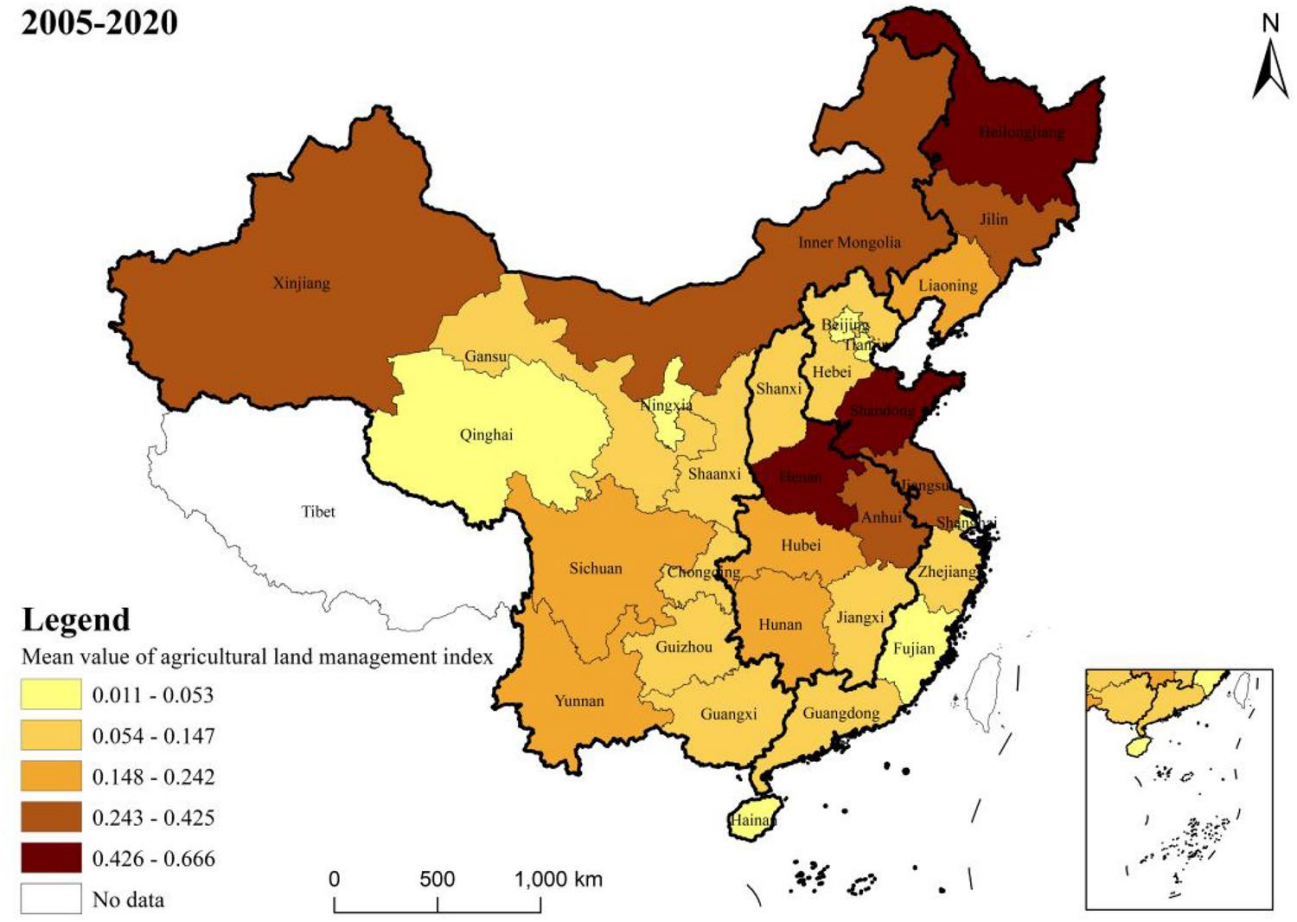
Mean value of agriculture land management index by province.
  Nationally, from 2005 to 2017, there was an upward trend in the national average level of farmland management, the average level of farmland management demonstrated a slow downward trend from 2017 to 2020. There are significant variations in agricultural management status among the eastern, central, western, and north-eastern regions. Among these, the northeastern region exhibited the highest farmland management index, with a mean value of 0.386, followed by the central region at 0.262, and a notable difference of 0.258 between the highest and lowest values.
  From the perspective of the provinces, at the provincial level, the disparities become even more pronounced. The top three provinces in terms of farmland management are Heilongjiang, Shandong, and Henan, with mean values of 0.666, 0.533, and 0.53 respectively. Conversely, Tianjin, Qinghai, and Shanghai rank as the bottom three, with mean values of 0.018, 0.013, and 0.011 respectively. The level of agricultural land management in all provinces and cities is improving comprehensively The fastest-growing provinces in this aspect are Heilongjiang, Inner Mongolia, and Qinghai, with average annual growth rates of 8.8%, 8.6%, and 8.3% respectively. However, Beijing and Shanghai experienced negative annual growth rates of −1.9% and −1.4% respectively. In terms of volatility, the eastern and central regions grew at a significantly slower rate compared to the western and northeastern regions.
2. Rural finance index
  Figure 5 presents the rural finance index measurements for each region in the country, while Fig. 6 shows the mean value of the rural finance index across 30 provinces.

**Figure 5:**
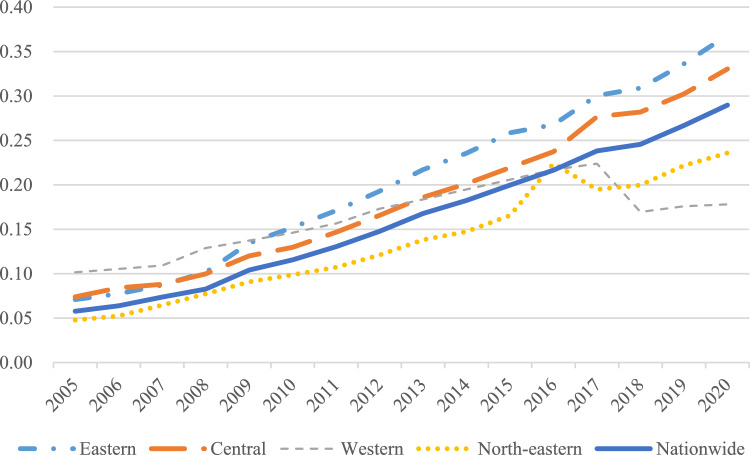
National and regional rural finance index.

**Figure 6:**
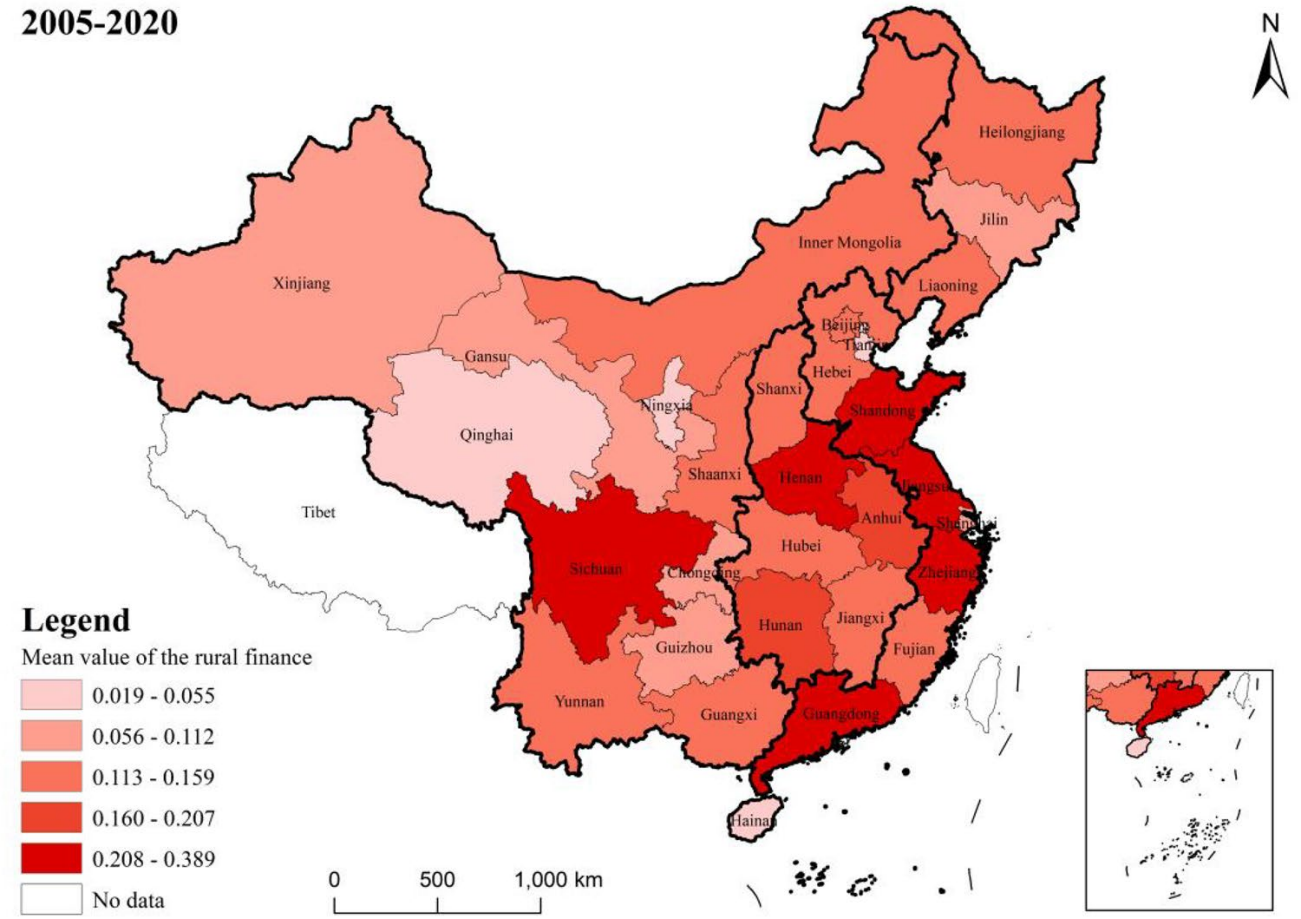
Mean value of rural finance index by province.
  Nationally, the level of rural finance has seen an annual growth both at the national and regional levels, from 0.058 in 2005 to 0.29 in 2020. However, despite this growth, the overall development level of rural finance is still relatively modest in numerical terms. At the regional level, there are significant disparities in rural financial development among the eastern, central, western, and northeastern regions. This is consistent with the results of Tan et al.^40^. The eastern region leads in terms of rural financial development with a mean value of 0.198, followed by the central and western regions with mean values of 0.205 and 0.116, respectively.
  When examining the rural financial development at the provincial level, there is a notable variation across provinces. The provinces of Guangdong, Shandong, Zhejiang, and Jiangsu are the top performers, with mean values of 0.389, 0.357, 0.355, and 0.345, respectively. These provinces are all located in the eastern coastal region. On the other side, Tianjin, Ningxia, Hainan, and Qinghai are ranked as the lowest, with mean values of 0.055, 0.024, 0.024, and 0.019, respectively. The rural finance index for each province and city demonstrates a fluctuating yet upward trend from 2005 to 2020, indicating a consistent year-on-year increase in the level of rural financial development in each province and city. The provinces having the fastest growth are Ningxia, Hainan, Qinghai, and Xinjiang, with average annual growth rates of 183.7%, 128.1%, 117.8%, and 104.4%, respectively.

#### Measurement and analysis of the degree of coordination of the coupling of agricultural land management and rural financial development

**Figure 7:**
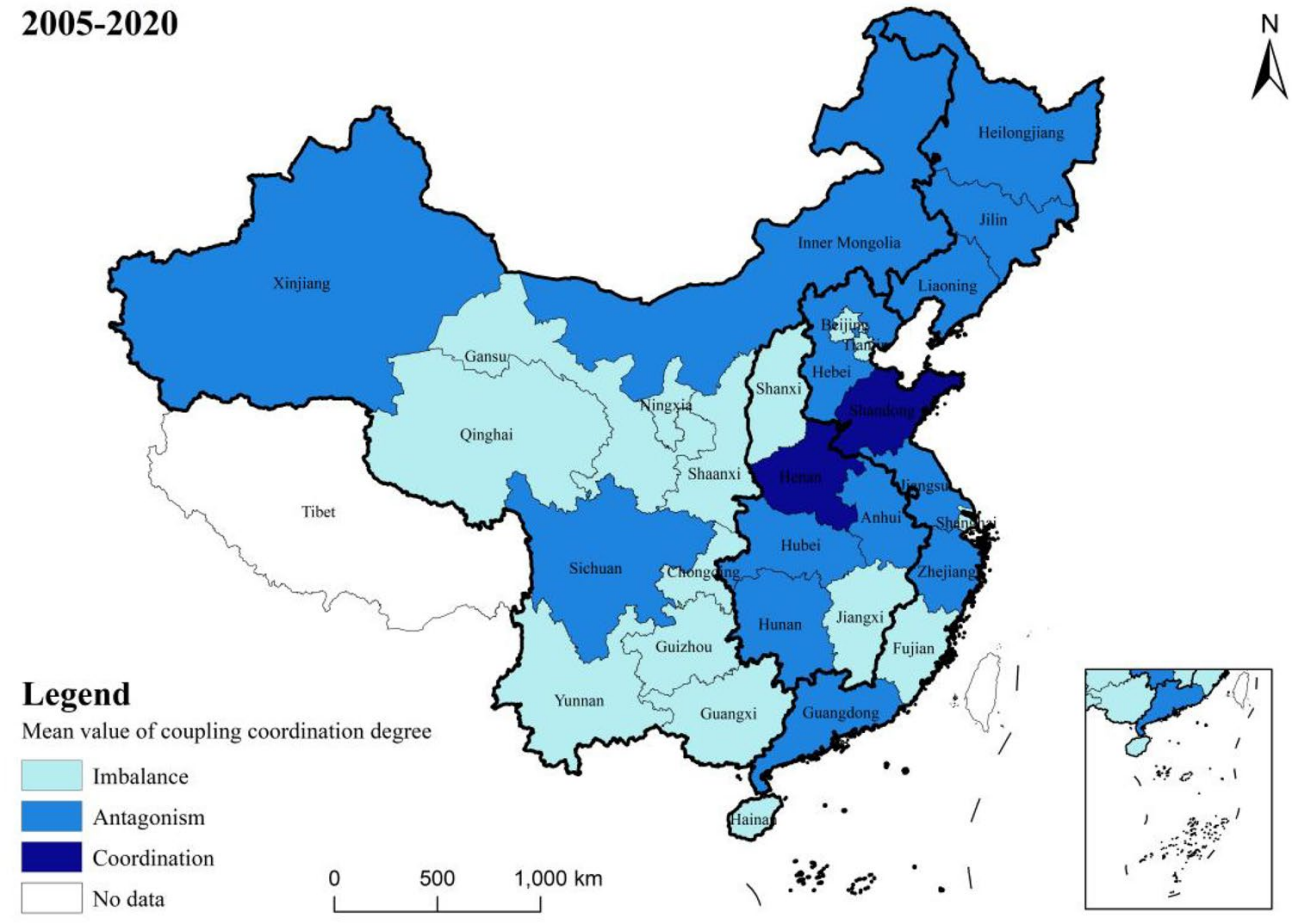
Mean value of coupling coordination by provinces.

**Figure 8:**
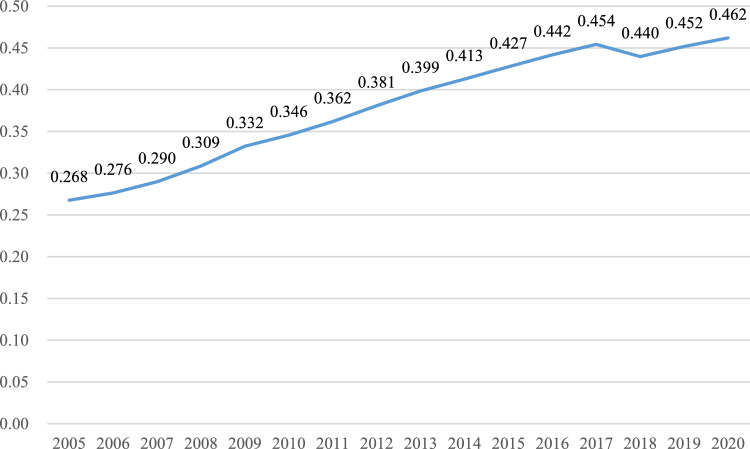
National mean value of coupling coordination from 2005 to 2020.

**Figure 9:**
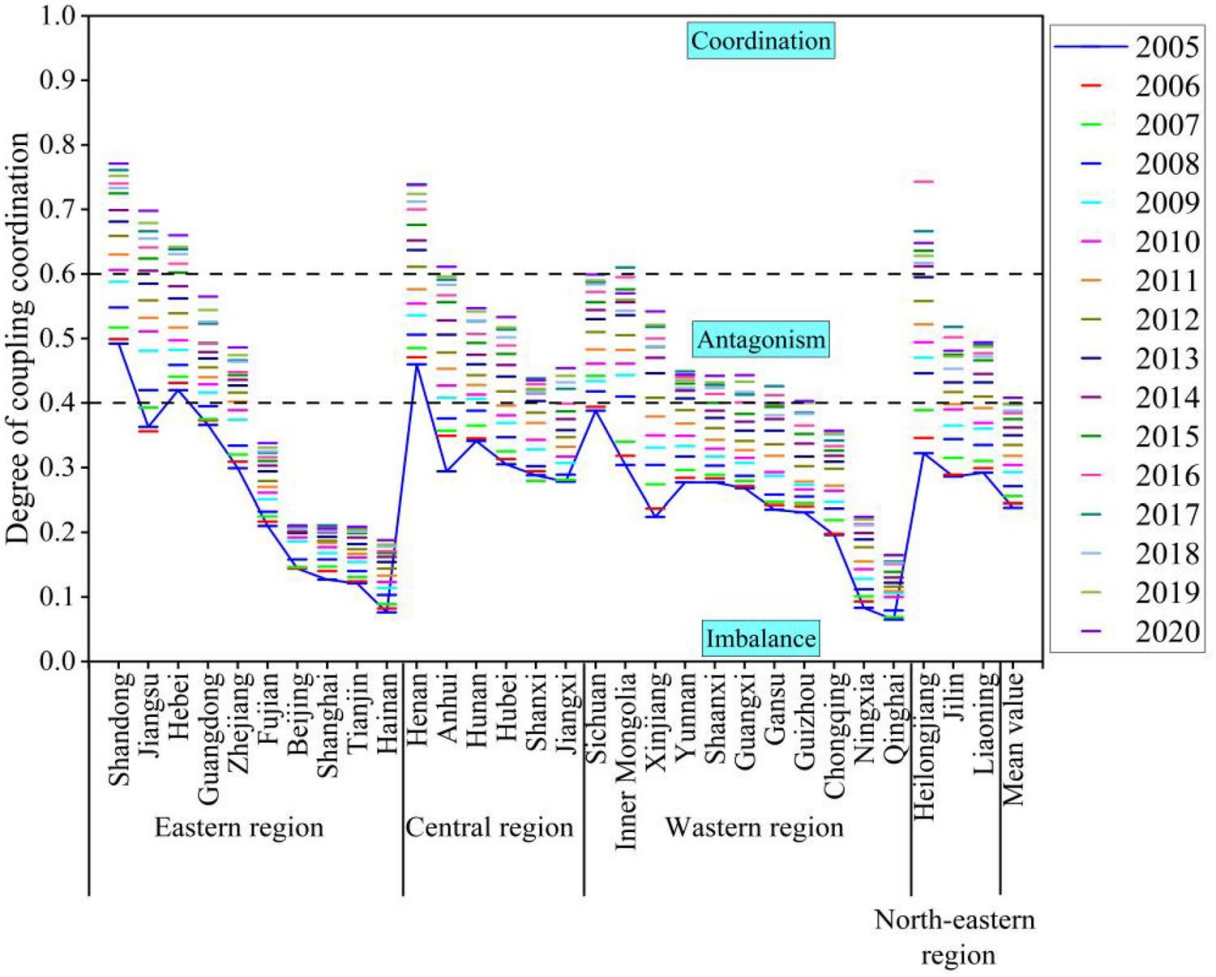
Degree of coupling coordination (D) and its mean value by province from 2005 to 2020.

Figure 7 illustrates the mean values of the coupling coordination degree of two systems (agricultural land management and rural finance) in each province from 2005 to 2020. Figure 8 presents the annual mean value of the coupling and coordination degree of these systems in 30 provinces across the country over the same period. Detailed measurement results are provided in Fig. 9.

From an overall trend perspective, the coupling coordination degree (D) of the farmland management and rural finance systems from 2005 to 2020 mainly ranges between 0.2 and 0.8. This range encompasses areas of imbalance, antagonism, and coordination, Temporally, the coupling coordination degree of agricultural land management and rural finance has shown a consistent year-by-year increase from 2005 to 2020. This suggests that these two systems are generally having a trend of mutual cooperation and coordinated development.

In terms of regional disparities, the Northeast has the highest mean value (0.454) for coupling and coordination between agricultural management and rural finance, which is considerably greater than the national mean value. There was a rising trend from 2005 to 2016, followed by a sporadic decline. The high degree of agricultural scale and increasing coverage and utilization of financial institutions in the three northeastern provinces have contributed to continuous growth in the rural financial market and subsequent transition to high-quality development. The Western region, despite an overall rising trend, has the lowest mean value (0.338) for coupling coordination. This is partly due to the lower land transfer rates and reluctance of farmers to transfer land in some Western provinces, and partly due to the low degree of urban–rural integration in this region, making it challenging for urbanization and rural construction to progress concurrently.

From the perspective of provinces, in 2005, the coordination degree of most provinces was categorized as middle dissonance and mild dissonance. By 2020, many provinces are in a state of primarily coordination, and intermediate level coordination. Similar to the views of Wang et al.^41^ and Liu et al.^42^, many provinces have shown a trend of coordinated development, a gap remains in achieving quality coordination. Challenges such as immature methods for confirming the rights to agricultural land have not yet fully matured, and problems such as high barriers to business entry, difficulties in collateral realization, unqualified collateral assessment, and inadequate risk mechanisms create impediments to the carrying out of business-rights mortgages. Shandong and Henan exhibit strong coupling coordination degrees, with mean values of 0.65 and 0.611. These regions use digital technology innovation as a core driver to provide financial support and risk protection for green agricultural development^43,44^. Conversely, Hainan and Qinghai Province have lower degrees of coupling coordination, at 0.14 and 0.118, respectively. Reflecting serious imbalances and uncoordinated development. The difference between the highest and lowest degrees of coupling coordination is 0.532, highlighting significant developmental and imbalance differences among provinces.

#### Analysis of types of coupled coordination

**Figure 10:**
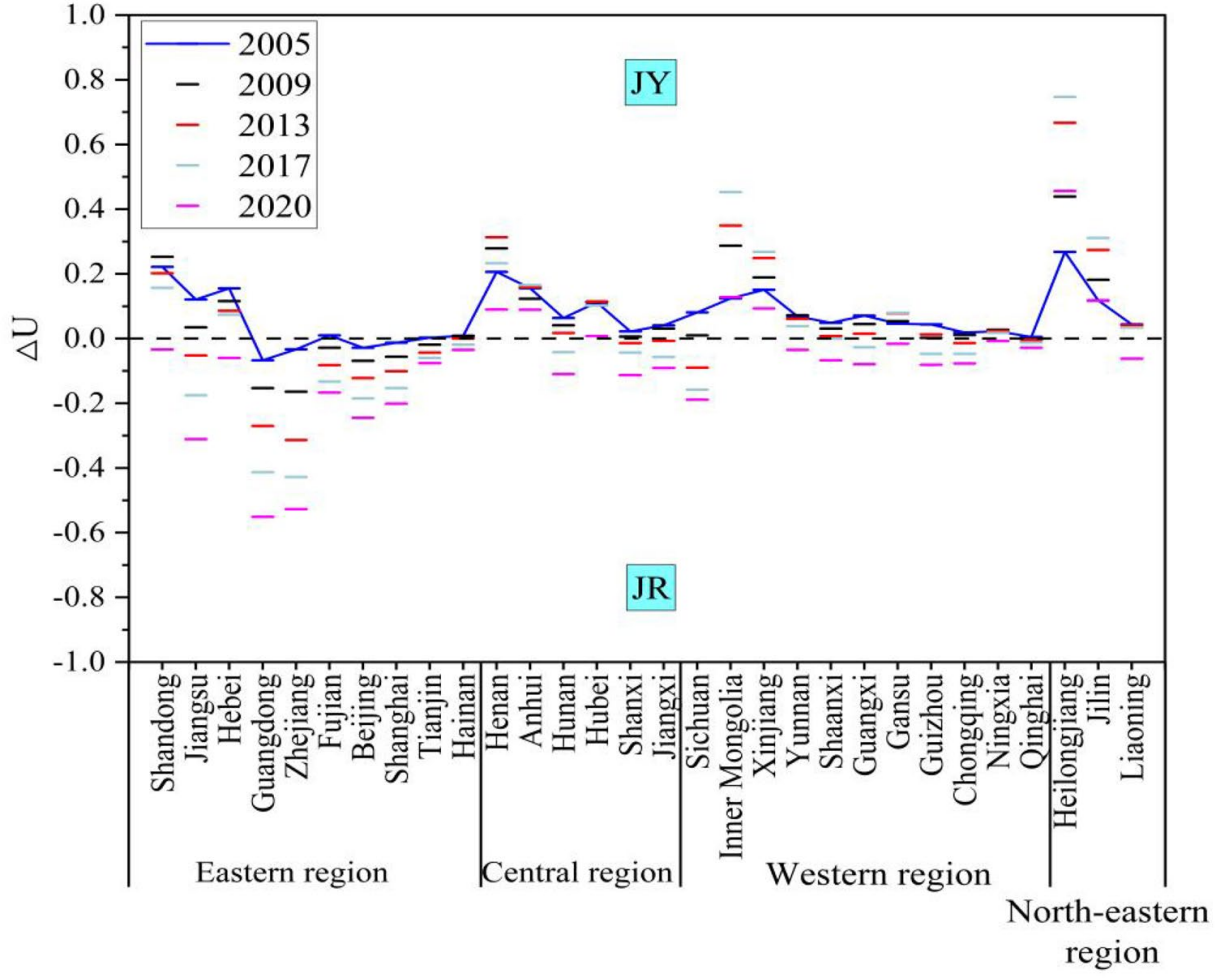
Types of coupled coordination by province.

Figure 10 presents a list of the types of coupled coordination between agriculture land management and rural finance in each province over a five-years period, selected at intervals. Among them, ${{\text{U}}}_{1}$ represents the value of the composite variable of agricultural land operation, and ${{\text{U}}}_{2}$ represents the composite variable value of rural finance. The coordination types are categorized based on the difference ($\Delta {\text{U}}={{\text{U}}}_{1}-{{\text{U}}}_{2}$). Combining existing scholarly research on the relative developmental advantages of subsystems^45,46^. This paper distinguishes two coordination types: when $\Delta {\text{U}}>0$ the corresponding year and province fall under the coordination type known as agricultural land management is the agricultural land management superiority type (JY); when $\Delta {\text{U}}<0$, the corresponding year and province coordination year and province coordination type is rural financial superiority type (JR).

Divided agriculture land management superiority type and rural financial superiority type. By exploring the relative development speed of subsystems, it is helpful to clarify the development advantages and financial support of agriculture land management in different regions, so as to optimize resource allocation and formulate more reasonable and effective agricultural production and financial policies according to the actual situation of each region, so as to make agriculture land management and rural finance more coordinated, thus promoting agricultural modernization and rural economic development.

In terms of the time span from 2005 to 2020, provinces and municipalities across the country generally exhibit ∆U year by year. There is a tendency for the growth of rural finance to outpace the growth of agricultural land management. This is especially evident given the rapid development of rural financing, where the gap between the two systems is gradually widening. This trend indicates that the current development of rural finance is mainly focused on 'quantity'. Consequently, there is an emerging phenomenon of inefficient use of agricultural funds. Moreover, the effectiveness of guiding farmers to operate farmland through rural financial tools is not enough, and its role in driving the large-scale operation of agriculture is insufficient. Consistent with the view of Long and Qu^2^, if the lack of a close link between rural financial funds and farmland management may, in the long term, affect the pace of large-scale agricultural management.

In terms of spatial span, Beijing, Shanghai, Zhejiang, and Guangdong consistently exhibit a negative ∆U, indicating they are always of the 'rural financial superiority' type (JR). These provinces, located in the eastern region, benefit from locational, talent, and policy advantages, and the development of rural finance here has been boosted by the overall economic operation trend. In contrast, Inner Mongolia, Jilin, Heilongjiang, Anhui, Henan, Hubei, and Xinjiang have always shown a positive ∆U, categorizing them as 'agricultural land management superiority' type (JY). Inner Mongolia and Xinjiang, for instance, suffer from a lack of geographic advantages and have experienced the issues like the loss of agricultural funds. Meanwhile, Jilin, Heilongjiang, Anhui, Henan, and Hubei, despite their high overall level of economic development, have large areas of farmland and significant populations, resulting in relatively low coverage and accessibility of financial institutions and services. Notably, the ∆U values of Inner Mongolia, Jilin, Heilongjiang, Anhui, Henan, Gansu, and Xinjiang demonstrate a trend of first increasing and then decreasing. This suggests that these provinces initially prioritize the development of agricultural land management before gradually accelerating the pace of rural financial development^47^.

### Spatial and temporal differences and dynamic evolution of the coupled development of agricultural land management and rural finance in China

#### Spatial and temporal differences in the development of the coupling of farmland management and rural finance in China

Measuring the degree of coordination of the coupling of agricultural land management and rural finance in China is analysed from an overall perspective, but since there are significant differences between different regions, it is necessary to further analyse the situation from a regional perspective.With reference to the current division of China's regions by most scholars^31,32^, we divide China into four regions: eastern, central, western, and northeastern. On the one hand, this will help to make comparisons between regions, discover the strengths and potentials of each region, and understanding whether there is an inequitable allocation of agricultural land management and rural financial resources; on the other hand, it will help the government and relevant institutions to formulate targeted policies, thus promoting the coordinated development of farmland management and rural finance.

In order to describe the spatial differences in the coupled development of the two systems and their sources, this article uses the Dagum Gini coefficient; Table 4 displays the measurement findings.

**Table 4:** Gini coefficient and decomposition results of the coupled development of agriculture land management and rural finance in China, 2005 to 2020.

Year	Totally	Intra-regional Gini coefficient				Inter-regional Gini coefficient						Contribution rate		
		Eastern	Central	Western	North–eastern	East–central	East–west	Central–west	East–northeast	Central–northeast	West–northeast	Intra-regional (%)	Inter-regional (%)	Hypervariable density (%)
2005	0.2215	0.2969	0.0916	0.2059	0.0265	0.2389	0.2843	0.1982	0.2272	0.0704	0.1622	27.54	32.88	39.58
2006	0.2185	0.2914	0.0928	0.2011	0.0408	0.2338	0.2772	0.2015	0.2220	0.0778	0.1645	27.35	34.40	38.25
2007	0.2198	0.2883	0.1047	0.2055	0.0522	0.2349	0.2758	0.1954	0.2210	0.0914	0.1778	27.62	31.01	41.37
2008	0.2169	0.2837	0.1036	0.1996	0.0662	0.2324	0.2681	0.1904	0.2238	0.0960	0.1889	27.38	32.13	40.49
2009	0.2107	0.2779	0.1006	0.1877	0.0616	0.2269	0.2621	0.1837	0.2178	0.0926	0.1814	27.29	31.92	40.79
2010	0.2116	0.2759	0.1003	0.1930	0.0665	0.2252	0.2621	0.1854	0.2168	0.0929	0.1870	27.39	32.05	40.56
2011	0.2094	0.2750	0.0979	0.1915	0.0660	0.2246	0.2587	0.1822	0.2175	0.0908	0.1838	27.45	31.72	40.83
2012	0.2079	0.2764	0.1008	0.1852	0.0710	0.2274	0.2556	0.1768	0.2210	0.0948	0.1797	27.42	30.54	42.05
2013	0.2081	0.2762	0.1025	0.1861	0.0747	0.2284	0.2539	0.1755	0.2245	0.0983	0.1818	27.44	29.97	42.59
2014	0.2057	0.2746	0.1001	0.1833	0.0734	0.2275	0.2515	0.1721	0.2238	0.0956	0.1789	27.42	29.75	42.83
2015	0.2053	0.2745	0.1035	0.1801	0.0717	0.2287	0.2498	0.1715	0.2252	0.0968	0.1796	27.31	30.18	42.51
2016	0.2100	0.2768	0.1044	0.1787	0.1029	0.2318	0.2493	0.1699	0.2461	0.1175	0.2039	26.72	31.95	41.33
2017	0.2062	0.2785	0.1061	0.1762	0.0698	0.2347	0.2515	0.1717	0.2274	0.0975	0.1752	27.12	30.23	42.65
2018	0.2038	0.2772	0.1032	0.1677	0.0710	0.2320	0.2520	0.1730	0.2231	0.0974	0.1597	26.98	30.67	42.35
2019	0.2028	0.2795	0.1026	0.1656	0.0654	0.2328	0.2517	0.1687	0.2237	0.0950	0.1574	27.06	29.69	43.25
2020	0.2045	0.2807	0.1006	0.1680	0.0688	0.2336	0.2542	0.1703	0.2256	0.0956	0.1609	27.02	29.93	43.06
Mean value	0.2102	0.2802	0.1009	0.1859	0.0655	0.2308	0.2599	0.1804	0.2241	0.0938	0.1764	27.28	31.19	41.53

1. Totally and intra-regional variations
  The overall and intra-regional variations in the evolution of the relationship between agricultural management and rural finance in China are depicted in Fig. 11a. In terms of the overall scope, there was a varying decreasing tendency in the total difference in the two systems' levels of coupling over the sample study period. The overall Gini coefficient's mean value was 0.2102, declining from 0.2215 in 2015 to 0.2102 in 2020 at an average annual rate of 0.51%. This suggests a minor narrowing of the gap in China's growth between the coupling of overall farmland management and rural finance.

**Figure 11:**
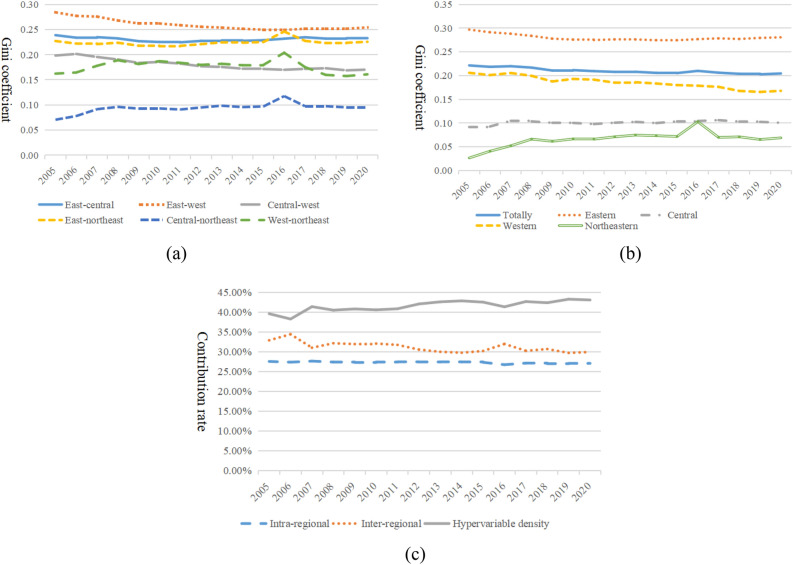
The size and contribution of spatial and temporal differences in the coupled development of agricultural land management and rural finance in China. (**a**) Totally and intra-regional Gini coefficients. (**b**) Inter-regional Gini coefficients. (**c**) Contribution of sources of regional disparities.
  Regarding intra-regional differences, differences in the synergistic development of agriculture land management and rural finance are most pronounced in the eastern provinces. This may be attributed to the fact that some Eastern provinces, such as Shandong, Jiangsu, and Hebei, lead the country in the development of agricultural land finance due to the rapid development of the rural economy. These areas also have favorable natural geographic environments and have achieved a high level of large-scale operation, resulting in a relatively high level of coordinated development between farmland operation and rural finance. However, provinces like Beijing, Shanghai, Tianjin, and Hainan have minimal agricultural land and their economies do not depend heavily on agricultural operations, leading to a lower degree of coordination between rural finance and agricultural land management. Consequently, significant differences have emerged in the coordinated growth of agricultural operation and rural financing among the Eastern provinces. The largest increase in the Northeast region may be due to the long-term lack of national strategic support for rural finance in this area. Despite the 2003 strategy of 'revitalizing the old industrial bases in the Northeast,' the financial issues in the region have not significantly improved. The differences in industrial structure, uneven capital investment, and varying degrees of openness to the outside world among the three eastern provinces have led to an increase in the differences in the coupled development of the two systems. In contrast, in the Eastern and Western regions, the Gini coefficients for the combined development of agricultural land management and rural finance exhibit a varying decreasing tendency. From 2005 to 2020, the average annual drop rates of the Gini coefficients were 0.36% and 1.23%, respectively, showing a decline in intra-regional disparities. Initially, the traditional rural financial path heavily depended on geographic location, transportation resources, regional development level, and other factors, leading to significant disparities in rural financial growth across provinces and municipalities. However, Similar views to Wang^48^, with the advent of intelligent, digital high-tech solutions, rural finance has become less reliant on traditional financial outlets and infrastructure. This technological shift, breaking geographical limitations and facilitating increased fund exchanges, has led to a simultaneous increase in the level of rural financial development and a natural narrowing of regional differences.
2. Inter-regional differences
  Figure 11b illustrates the evolution of inter-regional differences in the coupled development of agricultural land management and rural finance in China. Horizontally, the largest inter-regional differences are between the East and the West, with a inter-regional Gini coefficient of 0.2599. The degree of differences in the coupled development decreases in the following order: between the East and the Center (0.2308), the East and the Northeast (0.2241), the Center and the West (0.1804), the West and the Northeast (0.1764), and the Center and the Northeast (0.0938). The Eastern region, benefiting from its natural geographical advantages, talent resources, and advanced development concepts, supports the coordinated development of agricultural land and finance. In contrast, the Western region, located inland, lacks geographical advantages for the development of rural financial services, faces a relative shortage of human resources, and has less advanced development concepts. Despite the implementation of the Western development strategy, the growth of non-agricultural sectors, and digital development, which have reduced the threshold for rural financial development, these measures have not significantly bridged the gap in rural financial development between the Eastern and Western regions in a short period.
  The Central and Northeastern regions show a varying rising trend in the inter-regional growth of the coupling of agricultural land management and rural finance throughout the study period, with an average annual increase of 2.38%. In contrast, the inter-regional disparities in the combined growth of rural finance and agricultural land management in the remaining areas exhibit a cyclical declining tendency. This means the coupling development level in regions with high and low development is converging towards the national average.
3. Sources and contributions of regional disparities
  Figure 11c illustrates the regional contribution rates of China's agricultural land management and rural financial coupling development. The variability in contribution sources reflects changes in the mechanisms causing disparities in the development of agricultural land management and rural financial coupling.
  In terms of trends in contribution rate changes, the overall intra-regional contribution rate curve is relatively flat with minimal changes. The interregional contribution rate rose slightly from 32.88% in 2005 to 34.4% in 2006, and then fluctuated down to 29.93% in 2020. It shows an evolutionary process of "slight increase—fluctuating decline". The hypervariable density reflects the contribution of the overlapping parts to the overall difference in the process of dividing the regions. The curve is symmetrical with that of the inter-regional contribution rate, decreasing slightly from 39.59% in 2005 to 38.25% in 2006, and then fluctuating up to 43.06% in 2020. It shows a "slight decrease—fluctuating increase" evolution.
  In terms of the magnitude of the contribution, hypervariable density has the highest average contribution, with a mean value of 41.53%. This is followed by the inter-regional contribution with a mean value of 31.19%, which is slightly higher than the intra-regional contribution (27.28%). This reflects the fact that the inter-regional cross-over has a more pronounced impact on the overall disparity.

#### Dynamic evolution of the coupled development of agricultural land management and rural finance in China

**Figure 12:**
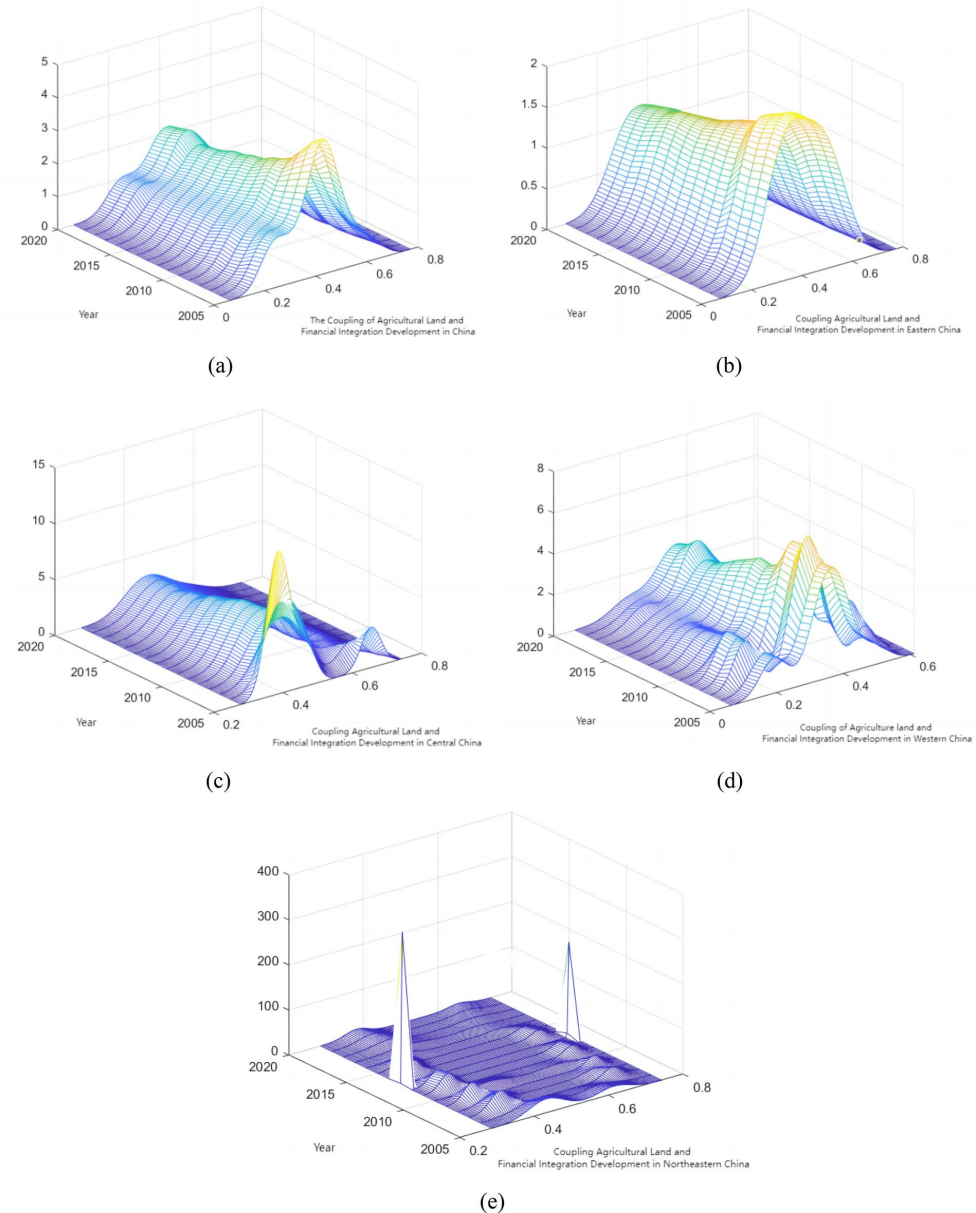
Dynamic distribution of coupled development of agricultural land management and rural finance in China and four regions. (**a**) National. (**b**) Eastern. (**c**) Central. (**d**) Western. (**e**) North-east.

To overcome the influence of uncertainty of unknown parameters, as well as to clarify the total distribution and change trend of the coupled development of agricultural land management and rural finance in different regions and time periods in China, this paper further uses the kernel density estimation method to depicts the shape and dynamic evolution law of the absolute difference distribution of the coupled development of the two systems, as shown in Fig. 12.

The combined growth of rural finance and agricultural land management in China is depicted through a dynamic kernel density distribution. As illustrated in Fig. 12a: first, the major peak of the distribution curve shifts consistently to the right, indicating an overall increase in the nation's level of combined agricultural land and financial development. Second, the initial decline and subsequent rise in the primary peak's height suggest that China's regional imbalance in the coupled growth of rural finance and agricultural land management often experiences a tendency of minor increase followed by a drop. Thirdly, it exhibits a 'single main peak with side peaks' trend, indicating a polarization phenomenon in the development of rural finance and agricultural land management. Over time, the two sides of the wave peaks progressively converge to the average level, suggesting a gradual improvement in the regional disparities in the development of these two fields.

The dynamic distribution of the kernel density of coupled development of agricultural land management and rural finance in the east is shown in Fig. 12b. From this figure, it can be observed that: first, the primary peak of the horizontal distribution curve consistently shifts to the right, demonstrating a progressive rise in the eastern region's degree of linked farming and financial growth. Second, the primary peak's height steadily declines over time, suggesting that the eastern area of China is experiencing a growing overall regional imbalance in the coupled growth of agricultural management and rural finance. Third, there is only one peak seen throughout the sample inspection time, indicating the absence of polarization.

Dynamic kernel density distribution in the combined growth of rural finance and agricultural land management in central China is illustrated in Fig. 12c: first, the primary peak of the coupling level distribution curve shifts entirely to the right, indicating a progressive rise in the center region's degree of linked financing and agricultural land development. Second, there is a noticeable downward tendency in the height of the major peak, suggesting that the regional imbalance in coupling development is progressively increasing. Thirdly, the movement of the wave peak from a double peak to a single peak—from a “single main peak and right peak” to a “single main peak”, indicates that the polarization phenomenon is waning with time. Fourth, there are varying degrees of narrowing of the gap between the average level of coupled development of the two systems in the central region and the lagging areas, as indicated by the right trailing phenomenon of the coupled development of agricultural land management and rural finance, which gradually converges over time.

The dynamic distribution of kernel density of coupled development of agricultural land management and rural finance in the west is presented in Fig. 12d. Here, it can be seen that: first, the coupling level distribution curve's primary peak shifts to the right overall, showing that the level of linked land and finance development in the west is continuously increasing. Second, the primary peak's height first declines and then rises, suggesting that the western region's regional imbalance in the coupled growth of rural finance and agricultural land management typically first declines and then slightly grows. Thirdly, the case of a 'single main peak with side peaks' is described; the left peak's height is less than the main peak's, suggesting a polarization phenomenon in the western region's growth of rural finance and agricultural land management. The shifted double peaks to the right show an improvement in the degree of development of the two systems' connection in the western area.

The dynamic distribution of kernel density of coupled development of farmland management and rural finance in Northeast China is shown in Fig. 12e: first, the main peak of the distribution curve of the coupling level shifts to the right as a whole, demonstrating that the level of the coupled development of farmland and finance in the Northeast has been increasing. Secondly, during the observation period, the existence of two main peaks in the Northeast region indicates a serious polarization phenomenon. Specifically, the growth rate of the coupling level of agricultural land management and rural financial development in Heilongjiang is much higher than that of Jilin and Heilongjiang, and the gap is increasing year by year.

### Decomposition of spatial effects of the coupled development of farmland management and rural finance in China

#### Global spatial autocorrelation

The Moran index is calculated to examine the spatial correlation between the levels of agricultural land and financial integration development each year. Table 5 shows that the global Moran indexes of the measured indexes of agricultural land and financial integration from 2005 to 2020 are all positive. The Z-value and P-value of these global Moran indexes further indicate, at the 10% level of significance, the presence of overall spatial autocorrelation in the development of agricultural land and finance integration among China's provinces and municipalities. This suggests the existence of inter-regional agglomeration in the integration development of agricultural land and finance.

**Table 5:** Values of the global Moran index for the integration of agricultural land and financial development.

Vintages	2005	2006	2007	2008	2009	2010	2011	2012	2013	2014	2015	2016	2017	2018	2019	2020
I	0.187	0.178	0.098	0.154	0.159	0.147	0.144	0.137	0.127	0.128	0.135	0.130	0.132	0.130	0.127	0.127
Z	1.998	1.909	1.182	1.682	1.721	1.613	1.587	1.525	1.433	1.444	1.503	1.476	1.473	1.459	1.435	1.430
P-value	0.023	0.028	0.119	0.046	0.043	0.053	0.056	0.064	0.076	0.074	0.066	0.070	0.070	0.072	0.076	0.076

#### Local spatial autocorrelation

**Figure 13:**
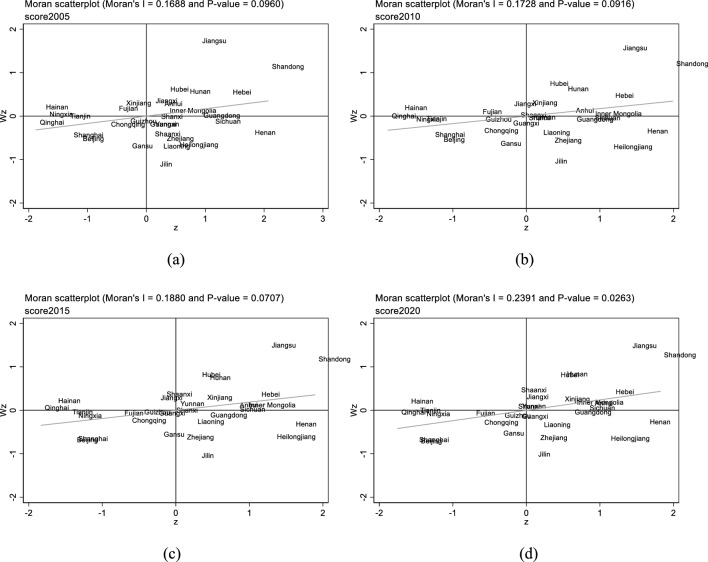
Spatial aggregation of synergistic development of agricultural land management and rural finance. (**a**) 2005. (**b**)2010. (**c**)2015. (**d**)2020.

In order to further explore the spatial pattern of the level of development of agricultural land and financial integration, here it is utilized the local Moran index for measurement. The analysis focused on four selected years, spaced at intervals between 2005 and 2020. The regional distribution of the local Moran index for the integration of agricultural land and financial development is presented in Fig. 13. The four quadrants in the table represent distinct spatial autocorrelation patterns.

As can be seen from Fig. 13, spatial agglomeration and heterogeneity characterize the development level of agricultural land and financial integration in China. Most regions fall into the first and third quadrants, signifying a dominance of high-high and low-low agglomeration, respectively. This pattern underscores a notable polarization. Predominantly, provinces in the first quadrant are situated in eastern and central China, while those in the third quadrant are mainly in the western region.

Provinces such as Hebei, Jiangsu, Anhui, Shandong, Henan, and Hubei exhibit higher levels of agricultural land and financial integration. Neighboring provinces also demonstrate elevated levels of coupled development, characterized by a high–high (H–H) agglomeration effect. This suggests a substantial influx of agricultural production factors and related funds into these areas, positioning them at the forefront of agricultural land and financial integration in neighboring provinces. This shift indicates a decline in the level of agricultural land and financial integration in Jilin, contrasting with higher levels in surrounding regions. This aligns with the earlier conclusion that the average coupled coordination value of farmland management and rural finance in Jilin is lower than the Northeast region's average from 2005 to 2020, with the disparity expanding annually. A significant factor is Heilongjiang's status as a leading grain-producing province with over 130 million mu of land transfers and advanced agricultural mechanization, propelling the development of large-scale farmland operations at a pace surpassing other Northeastern provinces. Fujian's transition from Quadrant 3 in 2005 to Quadrant 2 in 2010 signifies that while the level of farmland-finance integration in neighboring areas has improved, Fujian’s own level of integration remains relatively low. This shift reflects a dynamic interplay of regional development levels within the context of agricultural land and financial integration in China.

## Conclusions

The results of the study are as follows:

Firstly, the examination of specific facts reveals an increasing trend in the coupling and coordination between China's agricultural land management and rural financial development. This transition, moving from a dysfunctional to a harmonious state, shows progress, yet a significant difference between the two remains evident. Across provinces, the coupling level exhibits a "high in the east and low in the west" pattern, with varying degrees of coupling from one region to another.

Secondly, the disparity between the two systems in terms of coupling coordination is widening, with rural financial development surpassing agricultural land management. This indicates a real issue of inefficient agricultural fund utilization. While the central and western regions focus on agricultural land management growth, many eastern provinces are advancing in rural finance. The nature and sequence of development vary across regions.

Thirdly, the overall disparity between agricultural management and rural financial growth in China is showing a downward trend in regional variances. Hypervariable density, indicating increasing interaction between intra- and inter-regional differences, is the main source of overall disparities. The contribution of intra-regional differences to total disparities remains relatively stable, while that of inter-regional differences shows the decline.

Fourthly, the dynamic evolution of the coupled development of farmland management and rural finance in China reveals regional polarization^49^, particularly in central, western, and northeastern regions. Over time, regional differences are gradually diminishing. The peak of the coupling development distribution curve is shifting rightward, indicating continuous improvement in the coupling level. Moreover, there is significant spatial autocorrelation and uneven distribution of the coupling degree, with high aggregation effects primarily in eastern and central regions, and low aggregation in the western regions.

In light of these conclusions, the following policies are recommended:

Firstly, improving the property rights system for agricultural land. Clearly define the property rights of agricultural land and formulate a more flexible and market-oriented policy for the transfer of agricultural land, so as to increase the mobility and efficiency of the use of land resources. Increasing rural financial innovation and encouraging financial institutions to develop financial products and services that are suitable for agricultural characteristics. Stimulate the potential for coupled development of agricultural land management and rural finance in China. The rapidly developing eastern and northeastern regions should leverage science, technology, and capital to introduce diverse rural financial products tailored to regional capital needs, expanding financing channels. The less developed central and western regions should fully utilize land management right mortgage policies, establish robust agricultural mortgage platforms, and expanding the scope of new types of collateral such as land management rights and forest rights to achieve synergistic development.

Secondly, there should be emphasize on the mutual support between rural finance and farmland management systems. This involves encouraging the growth of innovative enterprises and enhancing the efficiency of financial investments in rural areas. Optimise and strengthen agricultural land use policies and agricultural support policies to increase the flexibility and efficiency of agricultural land use through the optimisation of land transfer markets and the clarification of land property rights. Concurrently, in the context of rapid development, rural finance should standardize agricultural fund approval processes to ensure funds are used efficiently. Government support rates for agriculture must also be guaranteed.

Thirdly, we have to focus on regional variations in rural finance and agricultural land management advancement. The Government should implement targeted regional pacification and development strategies, such as fiscal transfers and support measures for the development of special industries. Given the considerable regional disparities, it's crucial to enhance connections between these development areas. Regions should adapt rural finance development to local conditions, integrate regional cooperation into the policy system, break down development barriers, and achieve synergistic national development. Interregional dissemination and exchange of successful experiences and models of rural finance and agricultural land management should be promoted.

Fourthly, we must utilize regional advantages to counter the "Matthew effect". Optimising mechanisms for the allocation of funds and the rural financial system. Ensure that agricultural and rural development financing flows more to resource-poor and lagging regions, and narrowing funding distribution gaps caused by geographical, economic, and resource factors are essential. Strengthening interregional exchanges and cooperation, establishing regional cooperation mechanisms, such as inter-regional agricultural cooperation zones and rural financial services alliances, guiding the flow of agricultural production factors and capital from developed regions in the east and centre of the country to less-developed regions, and promoting technological exchanges, capital flows and the sharing of talents between developed and less-developed regions, promoting coordinated inter-regional development and reducing polarization.

## Data Availability

The datasets used and/or analysed during the current study available from the corresponding author on reasonable request.
